# Magnetic and Dielectric Properties of Nano- and Micron-BiFeO_3_/LDPE Composites with Magnetization Treatments

**DOI:** 10.3390/ma13010120

**Published:** 2019-12-26

**Authors:** Wei Song, Yu-Zhang Fan, Yu Hua, Wei-Feng Sun

**Affiliations:** Key Laboratory of Engineering Dielectrics and Its Application, Ministry of Education, School of Electrical and Electronic Engineering, Harbin University of Science and Technology, Harbin 150080, China; sw7912@hrbust.edu.cn (W.S.); yuzhang199672@163.com (Y.-Z.F.); huayu94111@163.com (Y.H.)

**Keywords:** low density polyethylene, bismuth ferrate, magnetization characteristics, dielectric permittivity

## Abstract

By means of magnetization treatments at ambient temperature and elevated temperatures, the nano- and micron-bismuth ferrate/low density polyethylene (BiFeO_3_/LDPE) dielectric composites are developed to explore the material processing method to modify the crystalline morphology, magnetic and dielectric properties. The magnetic field treatment can induce the dipole in the LDPE macromolecular chain which leads to preferred orientation of polyethylene crystal grains to the direction of the magnetization field. The surface morphology of the materials measured by atomic force microscope (AFM) implies that the LDPE macromolecular chains in BiFeO_3_/LDPE composites have been orderly arranged and form thicker lamellae accumulated with a larger spacing after high temperature magnetization, resulting in the increased dimension and orientation of spherulites. The residual magnetization intensities of BiFeO_3_/LDPE composites have been significantly improved by magnetization treatments at ambient temperature. After this magnetization at ambient temperature, the *M*_R_ of nano- and micron-BiFeO_3_/LDPE composites approach to 4.415 × 10^−3^ and 0.690 × 10^−3^ emu/g, respectively. The magnetic moments of BiFeO_3_ fillers are arranged parallel to the magnetic field direction, leading to appreciable enhancement of the magnetic interactions between BiFeO_3_ fillers, which will inhibit the polarization of the electric dipole moments at the interface between BiFeO_3_ fillers and the LDPE matrix. Therefore, magnetization treatment results in the lower dielectric constant and higher dielectric loss of BiFeO_3_/LDPE composites. It is proven that the magnetic and dielectric properties of polymer dielectric composites can be effectively modified by the magnetization treatment in the melt blending process of preparing composites, which is expected to provide a technical strategy for developing magnetic polymer dielectrics.

## 1. Introduction

Polymeric composites filled with nanoscale or micron inorganic particles possess specific nanostructure and excellent dielectric performance, which can be widely applied in various fields of an energy storage capacitor, magnetic information device, microwave absorption, etc. [1,2,3]. In particular, the polymer magnetic composites have been prominently applied in component separation technology, self-repairing materials and electronic nanodevices [4,5,6]. 

Employing inorganic nanofillers with magnetic moment as a magnetic source of composites, state-of-art dielectric and magnetic properties can be achieved to fulfill both magnetic response and polymeric advantages in prospective applications of medicine, environmental protection, electronic industry, electromagnetic interference shielding, etc. [7,8,9]. Especially, polymer-based composites with both dielectric and magnetic performances can be exploited to realize electronic devices with the advantages of miniaturization, lightweight and low cost, which has become a hot spot in industry and academia [10,11,12]. Nowadays, nanodielectrics (polymer dielectric nanocomposites) are developing towards functionalization and intelligence, highlighting the flexible designability of polymers. Therefore, it has become an important research field of dielectric composites to develop pertinent preparation technology and special processing methods.

Low density polyethylene (LDPE) has been extensively used as a dielectric material due to its excellent mechanical and electrical insulating performances, low cost and processing flexibility. Because bismuth ferrate (BiFeO_3_) represents the phase transitions of ferromagnetic and ferroelectric orders at a critical temperature higher than room temperature, it is unambiguously considered as a prospective candidate to realize magnetoelectric coupling at ambient conditions. The ferroelectricity and antiferromagnetism (multiferroicity) of BiFeO_3_ are prospective to be applied in the electric regulation of electromagnetic oscillations and the magnetic regulation of dielectric polarization. Magnetization is a new processing method of preparing polymer-based magnetic composites, by which the microstructure and crystallization can be changed to realize the microstructure modification. The properties of polymeric composites are primarily determined by the crystalline conformation of polymer molecules which is affected by the movement of molecular chains in crystallization process. Polyethylene is a representative, semicrystalline polymer with large degrees of freedom in the complicate molecular motions from the large scale displacements of polymeric macromolecules to the atomic-scale vibrations of molecular segments. The LDPE material is constituted by the co-existence of crystalline and amorphous configurations, in which the crystalline lamellars formed by periodically folding polyethylene macromolecular chains are diversely piled up into the spherulites being separated by the amorphous phase [13]. The dielectric performances of LDPE rely substantially on the crystallinity and morphological structures [14].

In the present study, we apply magnetic field treatments at room temperature and the melting temperature of LDPE in the processes of preparing nano- and micron-BiFeO_3_/LDPE composites to explore the magnetization effect on the crystalline morphology and magnetic property of BiFeO_3_/LDPE composites. The ultra-flat samples with atomic-scale plane surfaces are fabricated by using a mica cleavage plane as a film-pressing substrate. The crystalline morphology on the sample surface has been directly observed with resolved lamellars by an atomic force microscope (AFM) in the striking mode. BiFeO_3_/LDPE composites are also characterized by infrared (IR) spectrum tests to evaluate the crystallinity of the LDPE matrix. The magnetization curves and complex dielectric functions of BiFeO_3_/LDPE composites are tested to analyze the effects of applying a magetostatic field at different temperatures on the magnetic and dielectric performances of BiFeO_3_/LDPE composites.

## 2. Experiments

### 2.1. Material Preparation

**Figure 1:**
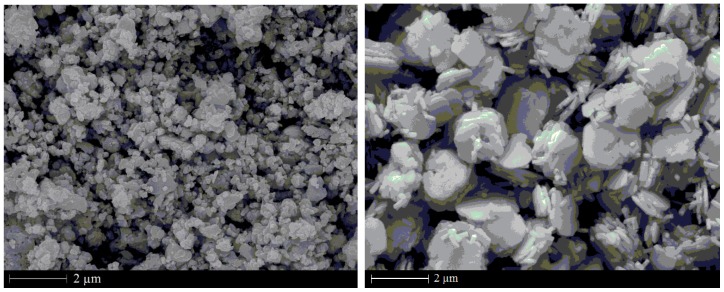
SEM images of the nanoscale particles (left panel) and micrometer-scaled multilayers (right panel) of BiFeO_3_.

Nanometer- and micrometer-scaled BiFeO_3_ particles which will be filled into LDPE are firstly synthesized by the hydrothermal route, in which the raw materials of ferric chloride (FeCl_3_) and bismuth nitrate pentahydrate (Bi(NO_3_)_3_·5H_2_O)·FeCl_3_ and Bi(NO_3_)_3_·5H_2_O are dissolved into glycol with the distilled water being added at a constant stirring rate until the solution has become a light yellow emulsion [15]. Then, the ammonia water is dropped into this emulsion so as to mediate the pH in 9–10. At last, the produced solid powders gathered by filtering are added into the sodium hydroxide solution in a reaction kettle, which will be heated for 24 h at 170 °C in the oven. The nanoscale particles and micrometer-scaled multilayers of BiFeO_3_ with the sizes of 100 nm and 2 μm, respectively, have been successfully prepared as illustrated by the images of scanning electronic microscopy (SEM) in Figure 1.

Nano- and micro-BiFeO_3_/LDPE composites are prepared by the two-step melt blending method: the suspension of BiFeO_3_ and LDPE is being melted and mixed in a torque rheometer at 140 °C for 30 min, and then the obtained melting material is placed into a plate vulcanizer being hot-pressed at 130 °C under a pressure of 14.5 MPa for 5 min. The film material in a thickness of 100 µm is finally achieved by extrusion. In the blending process of preparing BiFeO_3_/LDPE composites, the content of BiFeO_3_ particles is controlled according to a substantial proportion of 2 wt %.

For magnetization treatments, the film samples with diameter of 80 mm are magnetized by a constant stable magnetic field generator (SBV-220, Inpu Magnetoelectric Technology Development Co., Ltd., Changchun, China). The distance between the two magnetic poles with a diameter of 100 mm in SBV-220 can be adjusted from 0 to 70 mm. The uniform magnetic field with the direction perpendicular to pole surface can be applied by the maximum magnetic field intensity approaching to 2.8 T. Besides, the magnetic pole surface of SBV-220 can be heated to 200 °C so that the film samples being clamped between the magnetic poles of SBV-220 can be uniformly heated and magnetized. The film samples are applied by a steady and uniform magnetic field of 1.5 T with the field direction perpendicular to the film plane for 0.5 h at ambient and elevated temperatures, respectively, leading to magnetized samples named as R-M and H-M. For elevated-temperature magnetization, the sample is heated at heating rate of 5 °C/min to 130 °C and then cooled down at a rate of 5 °C/min to ambient temperature, in which the magetostatic field is always being applied.

### 2.2. Characterization and Test

The infrared absorption spectra are obtained from the transmission and attenuated total reflection being tested by Fourier transform infrared instrument (EQUINOX-55, Bruker AG, Karlsruhe, Germany) in the range of 400–4000 cm^−1^ with a spectral resolution of 4 cm^−1^. The absorption spectra are obtained by collecting transmitted intensity for 16 times of scanning.

The AFM tests are performed with a scanning probe microscope (Nano Scope IIIA, Bruker AG, Karlsruhe, Germany) to characterize the crystalline morphology of LDPE-based materials, in which the percussion mode with a scanning frequency of 1–2 Hz is adopted to output a surface height diagram. All experiments were carried out with the Pt conductive probe being coated with Si_3_N_4_ dielectric film material under ambient conditions (temperature of 25 °C and relative humidity of 25%).

Employing the SQUID magnetometer (MPMS-3, Quantum Design Inc, San Diego, CA, USA), the magnetic susceptibility is measured under direct current (DC)-applied magnetic fields ranging from −3 to 3 T at ambient temperature, in which a diamagnetic correction is applied for the sample holder. The alpha-A analyzer (Novocontrol, GmbH and Co. KG, Montabaur, Germany) is utilized to test 1–10^5^ Hz dielectric spectra at room temperature for the circular film samples with both sides being vacuum evaporated by aluminum film electrodes in 25 mm diameter.

## 3. Results and Discussion

### 3.1. Infrared Spectrum

**Figure 2:**
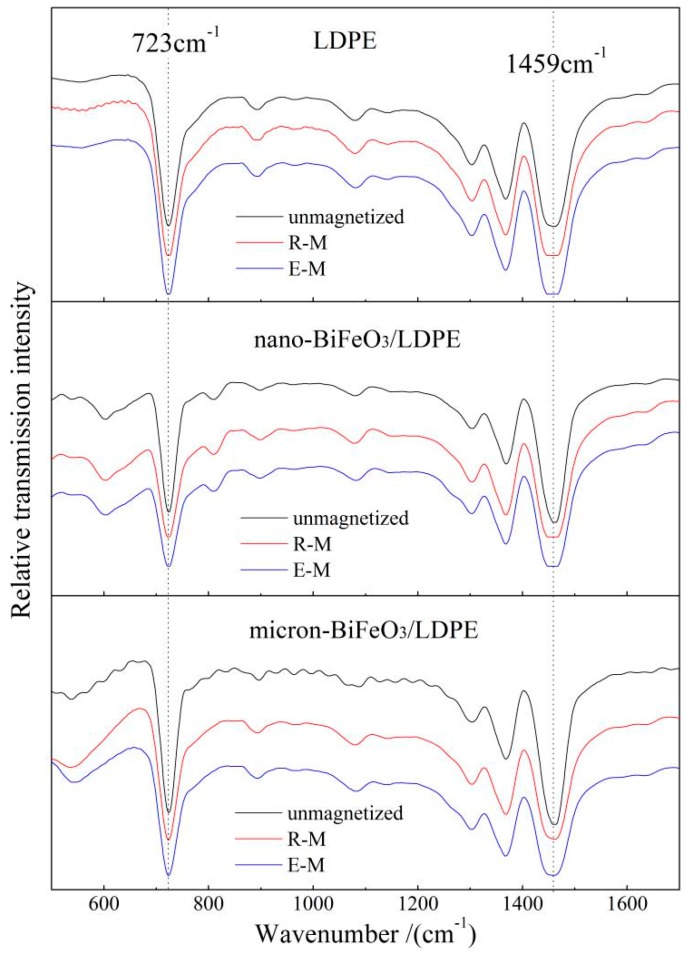
Fourier transform infrared (FTIR) spectra of low-density polyethylene (LDPE) (upper panel), 2 wt % nano-BiFeO_3_/LDPE composite (middle panel) and 2 wt % micron-BiFeO_3_/LDPE composite (bottom panel) after magnetization room (R-M) and elevated (E-M) temperatures.

The absorbance peak arising at 1459 cm^−1^ derives from the deformation vibration of molecular fragment −CH_2_−, while the characteristic sharp absorption at 723 cm^−1^ identifies the rocking motion of the larger atomic group −(CH_2_)_n_−, as presented by the infrared spectra of neat LDPE and BiFeO_3_/LDPE composites in Figure 2. In this present work, the orientation function of crystalline LDPE phases is calculated from the peaks at 723 and 1459 cm^−1^. The characteristic two peaks can simultaneously appear only when the crystalline polyethylene exists with a high concentration in the LDPE matrix, while only one single peak can be observed for the amorphous polyethylene system [16]. It is thus indicated from Figure 2 that crystalline phases with similar high content appear in all the BiFeO_3_/LDPE composites as well as in neat LDPE. Further, the magnetization effect on the molecular configuration of the LDPE matrix can be indicated by the intensity ratio of absorbance peaks in the infrared spectrum [17,18,19]. The ratio of peak intensity is defined as α = k *A*_723_/*A*_1459_ (k is a constant, *A* = lg(1/*T*) denotes the absorption coefficient, and *T* symbolizes the transmission coefficient) for the absorbance peaks locating at 723 cm^−1^ and 1459 cm^−1^ in the infrared spectra of neat LDPE and BiFeO_3_/LDPE composites, as the calculated results listed in Table 1. The higher α of magnetized samples than that of un-magnetized samples verifies that the magnetization treatment has increased the LDPE crystallinity especially at elevated temperature. The magnetization induces the dipole in the molecular chain which will alter the stress direction and increase the free energy of LDPE molecules. Therefore, the rocking and bending vibrations are enhanced and inhibited respectively by the magnetization treatments, leading to a preferred orientation of crystal grain with respect to the applied magnetic field direction, which accounts for the increased α of magnetized samples.

### 3.2. Crystalline Morphology

**Figure 3:**
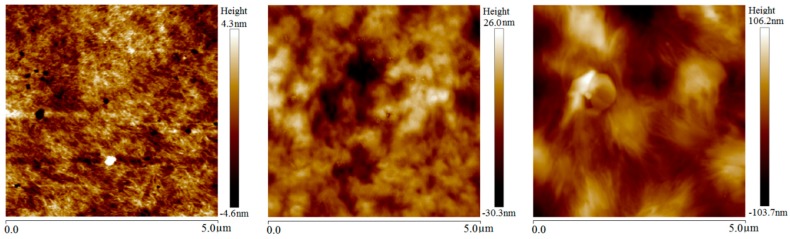
Atomic force microscopy (AFM) images of LDPE without magnetization (left panel) and magnetized at room (middle panel) and elevated (right panel) temperatures.

AFM height morphology images of LDPE film samples after different magnetization treatments are shown in Figure 3. The crystallization morphology of LDPE with magnetization treatments illustrates thicker lamellae and larger spacing between them than that of the unmagnetized sample, leading to appreciably larger spherulites. After being magnetized at room temperature, the lamella thickness and spherulite size increases slightly, as the blurred texture of lamellae shown in the middle panel of Figure 3. Because the folding orientation of macromolecular chains is still hindered in the process of elevated-temperature magnetization, the lamella thickness of LDPE increases obviously, while the inter-lamellar spacing decreases after magnetization, leading to enlargement of spherulites which are arranged in disorder without notable orientation. 

The complete crystallization process of polymer molecules goes through the initial molecular segments curling to form lamellar crystals in molecular-scale, the combining and twisting of lamellae in mesoscopic-scale and the aggregation of lamellars to form spherulites in the macro-scale [20]. Accordingly, the magnetic field force on flexible molecular chains of LDPE cannot produce the oriented lamellae, such as the cascade and straightening chain crystals, but can provide a tensile force to the folding molecular chains during the growing process of these lamellae, resulting in thicker lamellae and thus accounting for the increase of spherulite size and crystallinity. The whole chain and segments of polymer molecules can form orientation at discrepant conditions in the crystallization process: the segment orientation can be completed by the internal rotations around an ingle bond in the high elastic state of polymer; whereas, the whole molecular chain needs the cooperative movement of all molecule segments to realize an oriented structure, which can only be accomplished when polymer materials are being heated to the viscous flow state. Therefore, the crystallization morphology of LDPE has been evidently modified by the magnetization treatment at elevated temperature.

**Figure 4:**
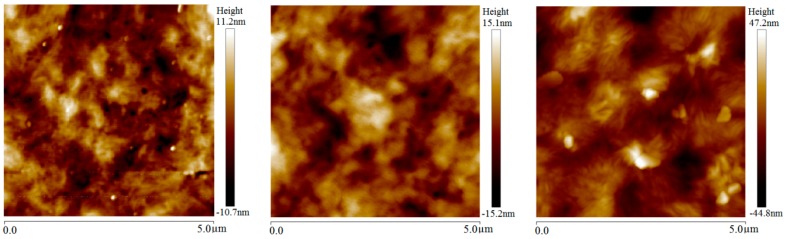
AFM images of 2 wt % micron-BiFeO_3_/LDPE composites without magnetization (left panel) and magnetized at room (middle panel) and elevated (right panel) temperatures.

**Figure 5:**
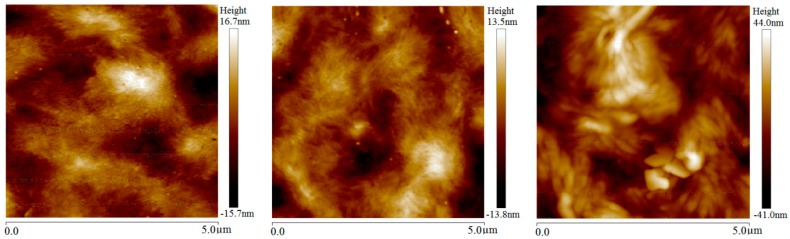
AFM images of 2 wt % nano-BiFeO_3_/LDPE composites without magnetization (left panel) and magnetized at room (middle panel) and elevated (right panel) temperatures.

The BiFeO_3_ micron-particles filled in LDPE play the role of inhomogeneous nucleating agents and remarkably increase the crystalline concentration of the LDPE matrix, as the notably larger spherulites of 2 wt % micron-BiFeO_3_/LDPE composites than that of neat LDPE compared by Figure 3 and Figure 4. Although the enlargements of crystal areas in the LDPE matrix are less obvious after room-temperature magnetization, the size and orientation of spherulites has been considerably increased by elevated-temperature magnetization due to more sufficient and complete crystallization from the intensified thermal movements of macromolecular chains. For polyethylene composites with a flexible molecular chain, the steric hindrance has little effect on the folding and crystallization of the whole polyethylene molecular chain at melting temperature. The multiferric BiFeO_3_ fillers in the LDPE matrix that have been magnetically polarized by magnetostatic field will engender tensile and compressive stresses on polyethylene molecules along and perpendicular to the magnetization direction, respectively, at room temperature, which leads to the increases of lamella thickness and spherulite size in BiFeO_3_/LDPE composites, as shown in Figure 4 and Figure 5. Due to the multiferric BiFeO_3_ nanoparticles filled into the LDPE matrix, which also act as a more efficient nucleating agent, the magnetic BiFeO_3_ nanofillers tend to be oriented under magnetic field in elevated-temperature magnetization treatment, and thus directly alter the crystalline conformation of the LDPE matrix in nano-BiFeO_3_/LDPE composites, as shown in Figure 5. Larger spherulites appearing in nanocomposites than micron-composites present a much clearer observation of filamentous lamellae aggregation. According to the slip diffusion model of nanodielectrics, the polyethylene molecules with intermediate phase near the surface of BiFeO_3_ nanofillers will be relaxed by the BiFeO_3_ orienting movement caused by magnetic polarization, and are prone to orderly rearrange at LDPE-melting temperature, eventually crystallizing on lamella surfaces to make lamellae thicker [21,22,23,24].

### 3.3. Magnetic Performance

**Figure 6:**
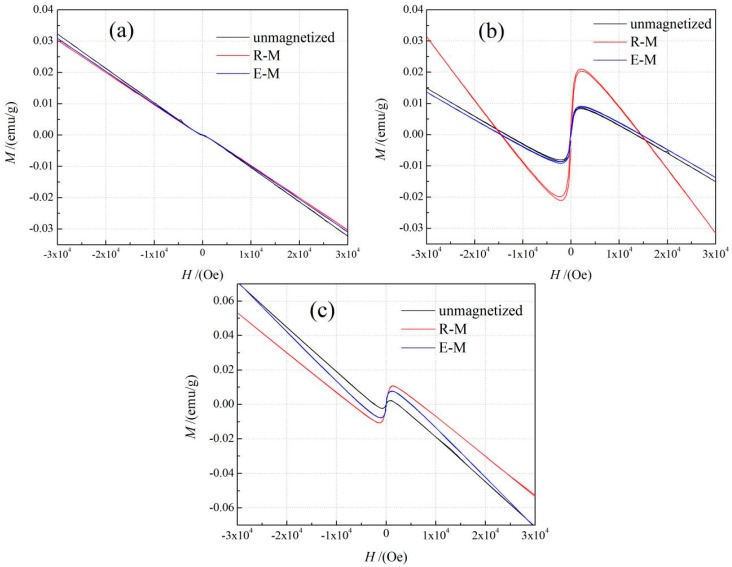
Magnetization curves of (**a**) LDPE; (**b**) 2 wt % nano-BiFeO_3_/LDPE composite and (**c**) 2 wt % micron-BiFeO_3_/LDPE composite after magnetization at room (R-M) and elevated (E-M) temperatures.

Under the action of an applied magnetic field, the magnetization *M* of LDPE decreases with the increasing magnetic field intensity *H*, with the magnetization direction being opposite to the magnetic field direction, as the magnetization curve of LDPE shown in Figure 6a. Furthermore, the negative magnetic susceptibility is very weak, even after the magnetization treatments at room or elevated temperature. The electron magnetic moments in the polyethylene molecule cancel out each other and cannot produce the net magnetic moment [12], as the inherent magnetization characteristics of diamagnetism LDPE shown in Figure 6a. After magnetization at room temperature and high temperature, LDPE represents the similar magnetization curves as that of the un-magnetized sample without any appreciable variation due to the diamagnetic characteristics with minimal negative susceptibility. The magnetization characteristics are remarkably changed after filling antiferromagnetic BiFeO_3_ particles into LDPE, as in the Figure 6b,c showing the magnetization curves of BiFeO_3_/LDPE composites prepared by different magnetization treatments. With the increase of applied magnetic field *H*, the magnetization *M* of BiFeO_3_/LDPE composites increases rapidly to a peak value, and then gradually decreases to a negative susceptibility similar to that of neat LDPE, which implies the transitions from antiferromagnetism to diamagnetism and the saturated process of BiFeO_3_ fillers in composites. The *M*-*H* curves of BiFeO_3_/LDPE composites exhibit antiferromagnetic and diamagnetic characteristics at low and high magnetic fields due to dominant contributions from BiFeO_3_ particles and the LDPE matrix, respectively. This promises that the magnetic behavior of LDPE can been significantly modified by filling antiferromagnetic BiFeO_3_ particles.

Parameters of magnetic properties including the tested maximum magnetization intensity (*M*_max_), residual magnetization (*M*_R_) and coercive force (*H*_C_) for neat LDPE, nano- and micron-BiFeO_3_/LDPE composites, are listed in Table 2. After magnetization treatments especially at room temperature, the nano- and micron-BiFeO_3_/LDPE composites acquire the higher residual magnetization *M_R_* of 4.415 × 10^−3^ emu/g and 0.690 × 10^−3^ emu/g, respectively. Because the size of BiFeO_3_ particles has a great influence on the magnetic properties of their composites, the weaker antiferromagnetism of the larger BiFeO_3_ fillers leads to a remarkably discrepant magnetic response of BiFeO_3_/LDPE composites [25]. The explicitly higher *M_R_* of nanocomposites than that of micron-composites can be attributed to the larger susceptibility caused by a more ordered magnetic moment in BiFeO_3_ nanoparticles than that in micron-particles. Due to the exacerbated thermal disturbance on magnetic order in BiFeO_3_ fillers at high temperatures, the composite samples with magnetization treatment at elevated temperature represent lower magnetization intensity than the composite samples with room-temperature magnetization treatment.

### 3.4. Dielectric Spectrum

**Figure 7:**
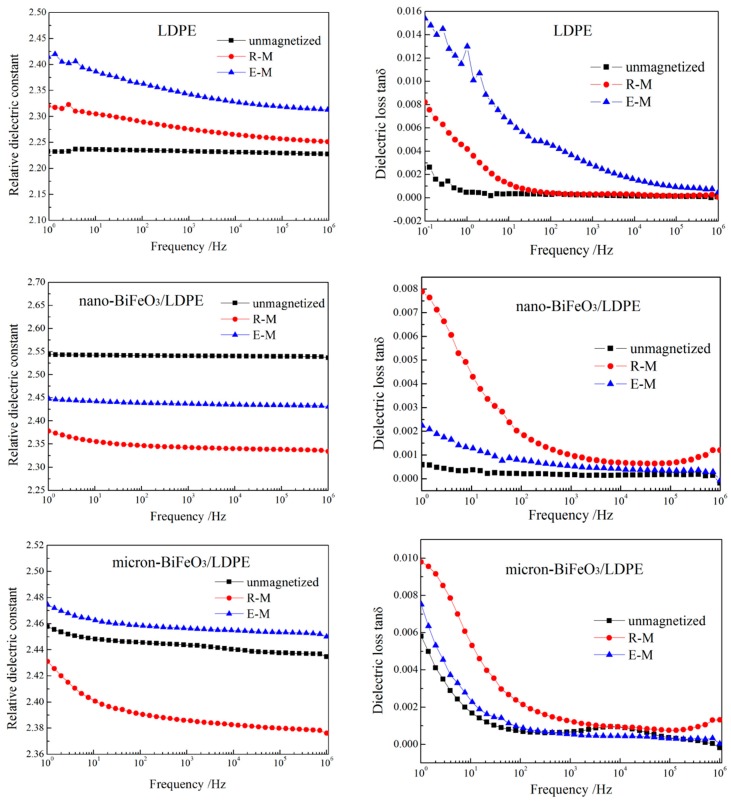
Complex dielectric spectra of LDPE (upper panels), 2 wt % nano-BiFeO_3_/LDPE composite (middle row panels) and 2 wt % micron-BiFeO_3_/LDPE composite (bottom panels) after magnetization treatments at room (R-M) and elevated (E-M) temperatures, for relative dielectric constant (left panels) and dielectric loss (right panels).

The dielectric performances of LDPE and BiFeO_3_/LDPE composites with magnetization treatments at room and elevated temperatures are investigated by testing complex dielectric spectra at the frequency range of 0–10^6^ Hz, as the results show in Figure 7. After magnetization treatments, the dielectric constant and loss of LDPE are apparently increased, especially with the highest increment being achieved by elevated-temperature magnetization, as shown in those upper panels of Figure 7. Polyethylene molecules are non-polar, atomic structures, which have neither weakly ionized ions nor polar groups, so that there is only electron displacement polarization under the action of an external electric field in LDPE. Therefore, the variations in molecular configuration and crystalline morphology caused by magnetization treatments will not substantially affect the dielectric constant of LDPE. The dielectric loss of LDPE originates primarily from impurity conductivity. In the process of elevated-temperature magnetization, the polyethylene molecular segments are rearranged with the impurities and structural defects of LDPE being excluded out of the spherulite boundary, leading to the increase of dielectric loss from the vibration and orientation movement of polar impurities in the amorphous region.

Because the dielectric constant of BiFeO_3_ particles is obviously higher than that of LDPE, the dielectric constant of BiFeO_3_/LDPE composites is slightly higher than that of LDPE. It can be deduced from the micron-scale magnetic domains in BiFeO_3_ material that BiFeO_3_ nanoparticles have only a single magnetic domain with a unified direction of spontaneous magnetization, while BiFeO_3_ micron-particles present multiple magnetic domains being randomly distributed in various directions. There is no turning-direction loss of magnetic domains in BiFeO_3_ nanoparticles under alternating electric field, so that nano-BiFeO_3_/LDPE composite (without magnetization) show similar dielectric loss as LDPE, as shown in the middle row panels of Figure 7. Whereas, the BiFeO_3_ micron-fillers in LDPE matrix will contribute to dielectric loss by turning direction of magnetic domain under alternating electric field, leading to a higher dielectric loss of BiFeO_3_/LDPE micron-composite than that of neat LDPE and BiFeO_3_/LDPE nanocomposite in low frequency range, as shown in bottom panels of Figure 6. The dielectric constant and loss of the magnetized nano-BiFeO_3_/LDPE composite are respectively lower and higher than that of the unmagnetized sample, as shown in the middle row panels of Figure 7. The dielectric constant reductions of BiFeO_3_/LDPE composites caused by magnetization treatment are attributed to the orientation of BiFeO_3_ fillers parallel to the magnetic field in the LDPE crystallization process under a magnetic field. The polarization of electric dipoles at the interfaces between BiFeO_3_ particles and the LDPE matrix will relax interface stresses which are produced by room-temperature magnetization, resulting in an appreciable increase of dielectric loss as shown in the right panels of Figure 7. In addition, it is not nearly for the BiFeO_3_ micron-particles with a minimal net magnetic moment to form orientation in melting LDPE, accounting for the negligible effect of elevated-temperature magnetization on the dielectric performances of the micron-BiFeO_3_/LDPE composite.

## 4. Conclusions

Dielectric BiFeO_3_/LDPE nano- and micron-composites are prepared with special magnetization treatments at ambient and elevated temperatures in the composite blending process. The effects of conditional magnetization on the crystallization morphology, magnetic properties and dielectric functions of BiFeO_3_/LDPE composites are analyzed to explore the magnetic modification technique in polymer dielectric composites. Under magnetization at ambient temperature, the microstructure, as shown by the crystallization morphology of the composites, suffers no considerable impacts from the magnetic field due to the slow peristalsis of macromolecular chains, and the magnetic and dielectric properties of BiFeO_3_/LDPE composites are dominantly attributed to the magnetic moments arrangement of multiferroic BiFeO_3_ particle fillers.

In contrast, under the magnetization at elevated temperature which is higher than the melting temperature of LDPE, the macromolecular chains are allowed to migrate preferably into highly efficient crystallization under the oriented actions of the BiFeO_3_ particles with the magnetic moment being polarized along the magnetic field direction, resulting in the increase of lamella thickness and spherulite size. BiFeO_3_ particles are easily arranged and oriented under the magetostatic field in the molten LDPE matrix, which improves the dispersion of BiFeO_3_ particle fillers. Therefore, after the elevated-temperature magnetization, the magnetic and dielectric properties of BiFeO_3_/LDPE composites are significantly affected by the crystallization morphology of the LDPE matrix and the magnetization of multiferroic BiFeO_3_ particle fillers. The present study suggests a feasible routine to simultaneously improve the magnetic and dielectric performances of magnetic-particle/polymer composites.

## Figures and Tables

**Table 1:** Infrared absorption coefficient and absorbance ratio.

Samples	*A*_723_ (a.u.)	*A*_1459_ (a.u.)	α (k)
LDPE	0.321	0.267	1.202
LDPE R-M	0.194	0.155	1.252
LDPE H-M	0.419	0.310	1.352
nano-BiFeO_3_/LDPE	0.056	0.076	0.737
nano-BiFeO_3_/LDPE R-M	0.229	0.201	1.139
nano-BiFeO_3_/LDPE H-M	0.276	0.215	1.284
micro-BiFeO_3_/LDPE	0.024	0.039	0.615
micro-BiFeO_3_/LDPE R-M	0.031	0.048	0.646
micro-BiFeO_3_/LDPE H-M	0.181	0.228	0.794

**Table 2:** Magnetic parameters of neat LDPE and BiFeO_3_/LDPE composites.

Samples	*M*_max_ (emu/g)	*M*_R_ (emu/g)	*H*_C_ (Oe)
LDPE	0.032	0.081 × 10^−3^	0.442
LDPE R-M	0.030	0.047 × 10^−3^	0.518
LDPE H-M	0.031	0.048 × 10^−3^	0.518
nano-BiFeO_3_/LDPE	0.015	1.932 × 10^−3^	0.455
nano-BiFeO_3_/LDPE R-M	0.031	4.415 × 10^−3^	0.341
nano-BiFeO_3_/LDPE H-M	0.014	1.956 × 10^−3^	0.341
micro-BiFeO_3_/LDPE	0.071	0.010 × 10^−3^	0.323
micro-BiFeO_3_/LDPE R-M	0.053	0.690 × 10^−3^	7.039
micro-BiFeO_3_/LDPE H-M	0.071	0.165 × 10^−3^	9.285
